# The Significance of Serum Interleukin-8 in Acute Exacerbations of Chronic Obstructive Pulmonary Disease

**Published:** 2018-01

**Authors:** Jingxi Zhang, Chong Bai

**Affiliations:** Department of Respiratory and Critical Care Medicine, Changhai Hospital, the Second Military Medical University, Shanghai, China.

**Keywords:** Exacerbation, Chronic Obstructive Pulmonary Disease, Interleukin 8, Interleukin 6, Tumor Necrosis Factor-α

## Abstract

**Backgrounds:**

Acute exacerbation of chronic obstructive pulmonary disease (AECOPD) is closely related to disease mortality. Systemic inflammation is considered to be involved in the pathogenesis of AECOPD. The current study aimed to investigate the clinical significance of the classic chemokine interleukin (IL)-8 in serum during AECOPD.

**Materials and Methods:**

In this current cross sectional, observational study, 50 patients with AECOPD, 25 patients with stable COPD and 25 healthy nonsmokers as the control group were selected. Clinical characteristics and spirometry data were collected. All patients were classified as grade 1–4 based on forced expiratory volume in 1 second (FEV1) after bronchodilation according to the GOLD severity classification and were divided into frequent exacerbation (FE) group (≥2 times/year) and non-frequent exacerbation (NFE) group (<1 time/year) according to acute exacerbation (AE) times in the previous 12 months before the visit. The serum IL-8, IL-6, tumor necrosis factor (TNF)-α, and superoxide dismutase levels were measured by the enzyme-linked immunosorbent assay technique.

**Results:**

Serum IL-8 levels increased sequentially from controls [9.45 pg/mL (ranged: 6.85–38.4)], to stable [51.60 pg/mL (ranged: 22.4–131.1)], and exacerbation stage [129 pg/mL (ranged: 57.7–374)]. The level of serum IL-8 was significant higher in patients with FE than that of patients with NFE (209.0 pg/mL (ranged: 115–472) vs 65.6 pg/mL (ranged: 11.2–149.3), P=0.008). A receiver operating characteristics curve (ROC) generated to evaluate IL-8, IL-6, and TNF-α levels to discriminate between patients with and without exacerbation showed that the total area under the curve (AUC) was 0.71 (95% confidence interval (CI): 0.5764–0.8381; P=0.003), 0.54 (95%CI: 0.4048–0.6943; P=0.54), and 0.52 (95%CI: 0.3912–0.6656; P= 0.7).

**Conclusion:**

Serum IL-8 is a sensitive, easy-to-measure, and inexpensive biomarker to give an indication of the course of COPD during exacerbation, and is a target to be explored further as a predictor to distinguish the patients prone to exacerbation.

## INTRODUCTION

Chronic obstructive pulmonary disease (COPD) is characterized by recurrent episodes of exacerbations defined as an acute worsening in the severity of patient’s symptoms, including dyspnea and coughing that warrants a change in medication or medical intervention. Acute exacerbations (AECOPD) are linked to disease-associated morbidity and mortality, placing significant strains on medical facilities, increasing resource burden, and driving up ongoing health care costs.

The mechanism of AECOPD is not elucidated comprehensively yet. AECOPD is considered as a kind of systemic inflammation besides the local airway inflammatory disease. The inflammation exaggeration induced by various pathogens or triggers was considered in the AECOPD, partly due to the “spillover” of such local inflammatory processes or the severity of the systemic inflammation itself (1). Several bio-substances and cytokines increase during the exacerbation such as C-reactive protein (CRP) (2), surfactant protein D (SP-D) (3,4), urokinase-type plasminogen activator receptor (suPAR) (5), interleukin (IL)-6, and TNF-α. The role of proinflammatory chemokines in AECOPD was studied on sputum or bronchoalveolar lavage fluid (BALF) samples, reflecting local condition. However, few researches focused on the role of the serum chemokines in AECOPD. Jónsdóttir et al., found that the serum level of the classic chemokine IL-8 can predict the mortality in patients with AECOPD and acute hypercapnic respiratory failure treated with noninvasive positive pressure ventilation (NPPV) within 28 days (6), but no other function of IL-8 in COPD was fully interpreted.

The current study aimed at investigating the clinical significance of the classic IL-8 in the COPD, especially in AECOPD.

## MATERIALS AND METHODS

### Study design

In the current cross sectional, observational study, a total of 50 patients with AECOPD and 25 patients with stable COPD were selected from the patients admitted to the Outpatient Department of Shanghai Changhai Hospital, China, with respiratory symptoms of progressive dyspnea, chronic cough and sputum production, a clinical diagnosis of COPD and demonstrated airflow obstruction on spirometry (JAGER MasterScreen lung function test machine, Germany) according to the 2005 American Thoracic Society (ATS) spirometry standards (7). Airflow obstruction was defined by a forced expiratory volume in one second to forced vital capacity (FEV1/FVC) ratio of <70% following the administration of 200 μg of inhaled salbutamol. The lung function grade of COPD was classified based on FEV1 as a predicted percentage (8). The 25 controls included asymptomatic never smokers who did not demonstrate any airflow limitation by spirometry. All subjects (cases and controls) were ethnically similar males. Smoking history was defined using pack-years. Exacerbations were defined as increase in any or all of the three major symptoms (dyspnea, sputum volume, and sputum color) from day to day routine in accordance with the definition of Burge and Wedzicha (9). All patients with AECOPD were classified based on GOLD severity classification as 1(≥80%), 2 (≥50% and <80), 3(≥30% and <50), and 4(<30%) grades according to the predicted FEV1 after bronchial dilation of each patient and were divided into frequent exacerbation (FE) (≥2 times) and non-frequent exacerbation (NFE) (<1 time) groups based on the occurrence of AE within 12 months before the visit. Blood collection and spirometry were performed at the same time in order to accurately correlate lung function with the levels of serum inflammatory mediators when presented for the first time in the hospital. The blood sample of each subject was stored in an EDTA (ethylenediaminetetraacetic acid) tube; supernatants (5 mL blood vein sample) were assayed for IL-8, IL-6, and TNF-α levels by the enzyme-linked immunosorbent assay (ELISA) technique (Diacolon, France) according to the manufacturers’ instructions, with a lower detection limit of 5, 4, and 2 pg/mL, and the upper normal limitations of 62, 5.9, and 8.1 pg/mL, respectively. All the supernatants were stored at −70°C until assay. Complete blood cell count (CBC) with differentiation was assayed for all the samples. Superoxide dismutase (SOD) level was assayed by ELISA based on the normal range of 129–216 U/mL. All the participants provided written informed consent. The study protocol was approved by the Ethical Committee of Shanghai Changhai Hospital in accordance with the Declaration of Helsinki.

### Statistical analysis

All data were analyzed using Graphpad Prism 5.0 or Sigmaplot 12.0 for windows. Continuous variables were expressed as mean ± standard deviation and median with interquartile range. Serum IL-8, IL-6, and TNF-α levels were transformed to a natural logarithm to mitigate the influence of extreme outliers. Differences between the two groups were evaluated by the Mann–Whitney U-test. Intergroup comparison of the three groups was conducted by the nonparametric Kruskal–Wallis test. Relationships between the serum levels of IL-8 and clinical variables were determined by univariate linear regression. Comparison between IL-8, IL-6, and TNF-α was performed by ROC (receiver operating characteristics curve) construction and the area under the curve (AUC) measurement. A P-value <0.05 was considered the level of significance.

## RESULTS

### Clinical characteristics

Totally, 100 male subjects including 50 patients with exacerbation of COPD, 25 patients with clinically stable COPD, and 25 healthy controls were included in the study. Their baseline characteristics are shown in Table 1. The three study groups were matched by age. By design, the results of pulmonary function test were normal in the control group and decreased in patients both in the stable COPD and AECOPD groups presented by spirometry values after bronchodilator inhalation and diffuse capacity for carbon monoxide of the lung (DLCO). Spirometry function significantly decreased in patients with exacerbation of COPD compared with the ones with stable COPD. The levels of antioxidative enzyme SOD reduced in both of the COPD groups, significantly.

**Table 1.** The clinical characteristics of the subjects included in the study

**Parameters**	**Control**	**Stable COPD**	**AECOPD**
Number	n=25	n=25	n=50
Age(years)	65.23±6.92	65.39±7.15	65.85±7.39
Smoking history(Pack years)	NA	39±17.5	40±19
BMI index	21.57±3.58	21.87±3.13	22.24±4.17
Blood neutrophil counts (×10^9^/L)	4.31±1.59	4.56±1.65	5.03±1.75
Blood neutrophil percentage (%)	60.30±8.44	58.93±7.89	65.61±8.9
Blood Eosinophil counts (×10^9^/L)	0.20±0.15	0.19±0.20	0.24±0.31
Blood Eosinophil percentage (%)	2.50±1.32	2.3±1.63	2.95±2.76
Post-FEV1/FVC(%)	83.44±5.53	52.23±14.21^**^	48.43±13.7^**#^
Post-FEV1(L)	2.50±0.61	1.84±0.95^**^	1.43±0.72^**^#
Post-FEV1%%predicted	116.17±17.12	65.03±31.35^**^	51.12±23.09^**^#
Post-FEF25–75(L)	2.45±1.45	0.81±0.68^**^	0.68±0.52^**^ #
Post-FEF25–75%predicted	106.38±24.82	24.85±19.53^**^	21.90±15.98^**^
Post-FEF50(L)	3.14±1.76	1.16±1.03^**^	0.85±0.75^**^#
Post-FEF50%predicted	116±27.30	28.13±24.10^**^	21.38±18.07^**^
DLCO (%)	91.97±21.01	63.66±6.44^*^	57.64±24.51^**^
SOD(U/ml)	173.52±27.68	143.62±12.08^*^	145.5±16.69^*^

**Note:**

* *P*<0.05 vs control group,

** *P*<0.01 vs control group,

# *P*<0.05 vs stable COPD group

**Abbreviations:** AECOPD, acute exacerbation of chronic obstructive pulmonary disease; FEV1, forced expiratory volume in one second; FVC, forced vital capacity; FEF, forced expiratory flow; DLCO, diffusion capacity for carbon monoxide of the lung; SOD, superoxide dismutase.

### Serum IL-8 was elevated in the exacerbation of COPD

**Figure 1.**
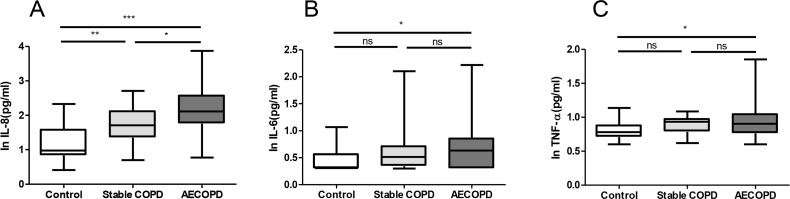
Comparisons of serum IL-8, IL-6 and TNF-α levels in controls (A), stable COPD (B) and COPD exacerbations (C).

As illustrated in Figure 1, the IL-8 levels increased sequentially from controls [9.45 pg/mL (ranged: 6.85–38.4)], to stable COPD [51.60 pg/mL (ranged: 22.4–131.1)] and COPD exacerbations [129 pg/mL (ranged: 57.7–374)]. The data were largely driven by differences in serum IL-8 levels between the controls and patients with COPD exacerbation (P <0.0001). IL-6 levels were 2.04 (ranged: 2.0–3.7), 3.27 (ranged: 2.2–5.1), and 4.29 (ranged: 2.0–7.16) pg/mL in the control, stable COPD, and AECOPD groups, respectively. TNF-α levels were 6.07 (ranged: 5.12–7.58), 8.59 (ranged: 6.1–9.43), and 8.05 (ranged: 5.8–11.10) pg/mL in the control, stable COPD, and AECOPD groups, respectively. The levels of IL-6 and TNF-α were higher than in the control group significantly (P =0.03 and P=0.018) (Figure 1)

### Serum IL-8 level in AECOPD was not influenced by pulmonary function grade

**Figure 2.**
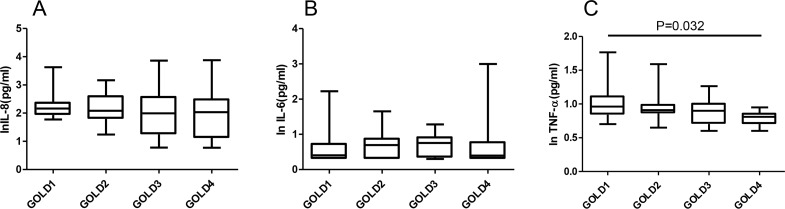
Comparisons of serum IL-8(A), IL-6 (B) and TNF-α (C) levels in different lung function grades of COPD exacerbations (Data was expressed in natural logarithm) **Note:** Box and whisker plots represent medians, interquartile ranges, and range.

There were 8, 12, 22, and 8 AECOPD cases with grades 1, 2, 3, and 4 based on Gold severity classification, respectively. The level of serum IL-8 in 1, 2, 3, and 4 grades were 146.5 (ranged: 86.6–236.3), 121.0 (ranged: 64.23–397.5), 98.7 (ranged: 18.3–375.0), and 108.0 (ranged: 14.18–322.3) pg/mL, respectively, with no significant differences between the groups. The serum level of IL-6 in 1, 2, 3, and 4 grades were 2.55 (ranged: 2.0–5.35), 4.97 (ranged: 2.0–7.5), 5.22 (ranged: 2.09–8.49), and 2.5 (ranged: 2.0–6.03) pg/mL, respectively, with no significant differences between the groups. The level of serum TNF-α in 1, 2, 3, and 4 grades were 9.13 (ranged: 6.96–12.98), 8.12 (7.23–9.68), 7.94 (5.06–10.19), and 6.68 (5.35–8.47) pg/mL, respectively, the level in grade 1 was higher than that of grade 4 (P=0.032) (Figure 2).

### Serum IL-8 level increased in patients with AECOPD and frequent exacerbation

**Figure 3.**
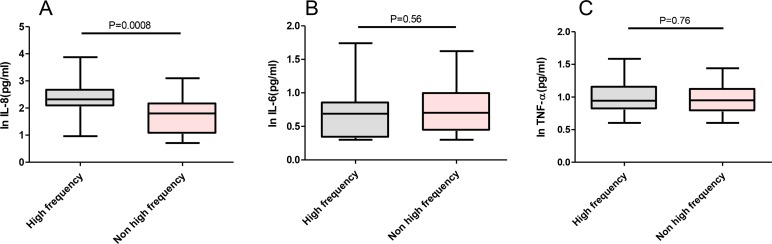
Comparisons of serum IL-8 (A), IL-6 (B) and TNF-α (C) levels in AECOPD patients with and without frequent exacerbation (Data was expressed in natural logarithm) **Note:** Box and whisker plots represent medians, interquartile ranges, and range.

There were 22 cases in FE and 28 cases in the NFE groups. The level of serum IL-8 was 209.0 (ranged: 115–472) pg/mL in patients with FE that was significantly higher than that of patients with NFE [65.6 pg/mL (ranged: 11.2–149.3)]. The level of serum IL-6 was 4.89 pg/mL (ranged: 2.14–7.17) in patients with FE and 5.05 pg/mL (ranged: 2.7–10.14) in patients with NFE, with no significant differences between the groups. The level of serum TNF-α was 8.76 pg/mL (ranged: 6.39–14.35) in patients with FE and 8.88 pg/mL (ranged: 6–13.1) in patients with NFE, with no significant differences between the groups (Figure 3).

### Variables associated with serum IL-8 in AECOPD

In patients with AECOPD, significant correlations were observed between serum IL-8 and blood eosinophil counts, blood eosinophil percentage, the change rate of predicted FEV1, DLCO, and SOD levels in Table 2.

**Table 2.** Pearson correlation coefficients of serum IL-8 levels with various clinical factors in patients with AECOPD

**Variables**	**r^2^**	**95%CI**	**P value**
Blood neutrophil counts	0.017	−0.0003481 to 0.0006630	0.524
Blood neutrophil percentage	0.003	−0.002923 to 0.002247	0.789
Blood eosinophil counts	0.1259	0.000005857 to 0.0001714	0.036
Blood eosinophil percentage	0.161	0.0001537 to 0.001588	0.018
The change rate of FEV1% predicated	0.09219	−0.005103 to −0.0002079	0.034
DLCO	0.1737	0.001417 to 0.01270	0.016
SOD	0.069	−0.006081 to −0.0002167	0.036

**Abbreviations:** AECOPD, acute exacerbation of chronic obstructive pulmonary disease; DLCO, Diffusion Capacity for Carbon Monoxide of the Lung; SOD, superoxide dismutase.

### Serum IL-8 was associated with other inflammatory mediators in AECOPD, but not in stable COPD

**Figure 4.**
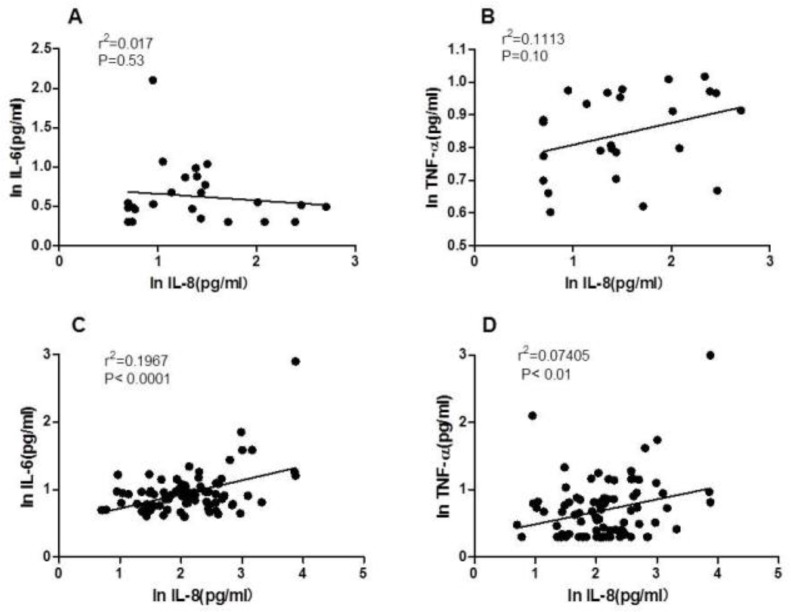
The relationships between serum IL-8 level and serum IL-6, TNF-α level in stable COPD (A,B) and COPD exacerbations (C,D) (Data was expressed in natural logarithm).

In AECOPD, there was a positive correlation between the serum level of IL-8, and the serum levels of IL-6 (r^2^= 0.1967, P<0.0001) and TNF-α (r^2^=0.074, P<0.0001). In stable COPD, the above relationship was not observed with IL-6 (r^2^=0.017, P=0.53) and TNF-α (r^2^=0.111, P=0.1) (Figure 4).

### Serum IL-8, but not IL-6 and TNF-α discriminate the exacerbation of COPD

**Figure 5.**
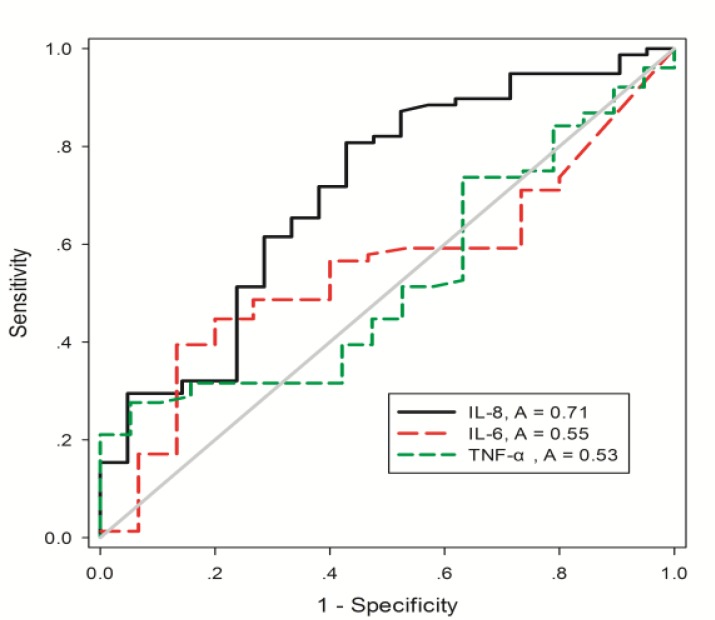
ROC curve for serum IL-8, IL-6 and TNF-α in discrimination of patients with acute exacerbation of chronic obstructive pulmonary disease.

ROC was generated to evaluate the ability of IL-8, IL-6, and TNF-α levels to discriminate between patients with and without exacerbation using all subjects in the current study. The total AUC was 0.71 (95% confidence interval (CI): 0.5764 – 0.8381; P=0.003), 0.54 (95%CI: 0.4048 – 0.6943; P=0.54), and 0.52 (95%CI: 0.3912 – 0.6656; P= 0.7). The most optimal cutoff point of sensitivity and 1-specificity was 82 mg/L for IL-8 in which sensitivity and specificity were 71.79% (95%CI: 0.6047 – 0.8141) and 61.9% (95%CI: 0.3844–0.8189), respectively (Figure 5).

## DISCUSSION

The current study found that the serum IL-8 elevated in patients with COPD, especially in acute phase with frequent exacerbation, and was closely associated with simultaneously elevated IL-6, TNF-α, blood eosinophils increase, and SOD reduction systematically, and also related with airway reversibility and diffuse capacity in the pulmonary part. Meanwhile, IL-8 showed the superior sensitivity compared with other two commonly used cytokines (IL-6 and TNF-α) to suggest the exacerbation of COPD.

The search for reliable biomarkers in COPD, other than FEV1, is always a research field of great interest to the clinicians. Specific biomarkers are useful to diagnose and monitor disease processes. IL-8 is a chemokine that induces the migration of neutrophils to the airway and affects degranulation produced by various kinds of cells. Both sputum and serum IL-8 are targets for research in regards to COPD mortality and exacerbations. Huang et al., found that plasma IL-8 increases in AECOPD and asthma-COPD overlap syndrome (ACOS) (10). A review by Koutsokera et al., summarized that sputum IL-8 levels in patients with AECOPD can predict clinical severity, symptomatic recovery, and even presence of bacterial infection and eradication conditions (11). Agusti et al., identified six inflammatory biomarkers (including IL-8) related to systemic inflammation, and reported that patients with COPD and elevated biomarkers are at high risk for increased all-cause mortality and exacerbation frequency during a three-year follow-up (12). The current study found that IL-8 as well as IL-6 and TNF-α is significantly elevated in exacerbations, which was similar to other study results, suggesting serum IL-8 as a common mediator of AECOPD in different countries. Here, there was an elevation in serum IL-8 in stable patients, compared with the controls, but not in IL-6 and TNF-α levels. Since many forms of acute and chronic illnesses are characterized by enhanced inflammation, it is likely that high baseline value of IL-8 reflects a more severe acute condition and most likely a more severe chronic illness.

In the current study, IL-6 and TNF-α were two well-known traditional systemic inflammatory mediators of COPD exacerbation; the finding was also reported by most previous studies (13). The comparison between IL-8 and the above two mediators in specificity and sensitivity of AECOPD was not reported before. The current study compared these three mediators of AECOPD and for the first time proved that serum IL-8 is more sensitive than IL-6 and TNF-α in Chinese population. The above results suggested that in AE stage the magnitude of IL-8 increase was more remarkable, and modulating IL-8 should be concentrated as the key target to treat AECOPD.

Predicting the response to therapy is another value for the biomarker to identify the individuals with COPD at higher risk of progression, guide more personalized management, and slow disease progression. Lastly, Shafiek et al., showed that in patients with AECOPD, serum IL-8 had higher levels in non-responders to NPPV vs. the responders (14). Jónsdóttir et al., found that IL-8 can predict the mortality of patients with AECOPD and hypercapnia respiratory failure within 28 days after the emergence of manifestations (6). The current study found that IL-8 increased significantly in the patients with highly frequent exacerbation. Hasegawa et al., found that patients with AECOPD with eosinophilia had higher frequency of readmission for AECOPD during a 1-year follow-up (15). Rahimi-Rad et al. also confirmed that the eosinophilia can predict the poor outcome of the patients with AECOPD including longer duration of hospitalization, need to mechanical ventilation, and higher in-hospital mortality (16). The current study found an association between IL-8 and blood eosinophils during the exacerbation; however, eosinophilia was not observed in the current study patients, which may be due to the small sample size. Although IL-8 was well-known to drive neutrophils recruitment and activation, Kawashima et al., found that IL-8 can induce eosinophils enrollment indirectly by neutrophil activation (17). Base on the abovementioned results, authors believe that IL-8 has the potential to predict the clinical outcomes in COPD. Authors are now conducting further studies to investigate whether or not IL-8 predicts frequent exacerbation in patients with COPD.

There was a close relationship between IL-8 and simultaneous elevation of IL-6 and TNF-α in exacerbation; however, this relationship was not observed in the stable stage. However, serum IL-8 had no relationship with airway obstructive severity. IL-8, not IL-6 or TNF-α, was still significantly higher in stable stages, suggesting that the inflammation induced by IL-8 occurred in the exacerbation process and also persisted in stable stage and illustrating the pathway of modulating IL-8 expression maybe different from that of the expression of IL-6 and TNF-α, which is worthy of further exploration.

Results of the current study showed the association between IL-8, and serum SOD level and some pulmonary function indexes including higher change rate of FEV1 values and reduced diffusing capacity. Oxidation and antioxidation imbalance due to SOD reduction was observed in all patients with COPD and was deteriorated in the exacerbation conditions that was similar to the results of other studies (18–20). Furuse et al., found that the elevated IL-8 level significantly correlated with decreased percentage of DLCO in systemic sclerosis maybe due to the fibroblast cell activation and endothelial impairment (21). Here, it was proposed for the first time that IL-8 maybe involved in both airway ventilation and gas exchange function. IL-8 increased mucus secretion by increasing the expression of genes encoding mucins (MUC5AC and MUCB) and resulted in the airway smooth muscle contraction (22,23), which explains the lung function deterioration in the current study patients with AECOPD. The current study does not allow any conclusions whether or not the relationship between IL-8 and the studied parameters was causal; however, it encourages further mechanistic studies to better understand the issue.

According to the results of the current study, it can be concluded that serum IL-8 can be a sensitive, easy-to-measure, and inexpensive biomarker to give an indication of the course of COPD during an exacerbation; and it is a target to be explored further as a predictor to distinguish the patients with COPD prone to exacerbation to implement individualized treatment and improve the disease outcome.
